# Effects of muscle fatigue on exercise-induced hamstring muscle damage: a three-armed randomized controlled trial

**DOI:** 10.1007/s00421-023-05234-z

**Published:** 2023-06-18

**Authors:** Carsten Schwiete, Christian Roth, Christoph Skutschik, Sebastian Möck, Lukas Rettenmaier, Kevin Happ, Holger Broich, Michael Behringer

**Affiliations:** 1 Department of Sports Sciences, Goethe University Frankfurt, Frankfurt am Main, Germany; 2 Department of Exercise Science, Olympic Training and Testing Center of Hessen, Frankfurt am Main, Germany; 3 Department of Science, Performance and Fitness, FC Bayern München AG, Munich, Germany; 4 Department of Performance, Neuroscience, Therapy, and Health, Medical School Hamburg, Hamburg, Germany

**Keywords:** Muscle damage, Muscle fatigue, Hamstring injuries, Damage markers

## Abstract

**Purpose:**

Hamstring injuries in soccer reportedly increase towards the end of the matches’ halves as well as with increased match frequency in combination with short rest periods, possibly due to acute or residual fatigue. Therefore, this study aimed to investigate the effects of acute and residual muscle fatigue on exercise-induced hamstring muscle damage.

**Methods:**

A three-armed randomized-controlled trial, including 24 resistance-trained males, was performed allocating subjects to either a training group with acute muscle fatigue + eccentric exercise (AF/ECC); residual muscle fatigue + eccentric exercise (RF/ECC) or a control group with only eccentric exercise (ECC). Muscle stiffness, thickness, contractility, peak torque, range of motion, pain perception, and creatine kinase were assessed as muscle damage markers pre, post, 1 h post, and on the consecutive three days.

**Results:**

Significant group × time interactions were revealed for muscle thickness (*p* = 0.02) and muscle contractility parameters radial displacement (D_m_) and contraction velocity (V_c_) (both *p* = 0.01), with larger changes in the ECC group (partial η^2^ = 0.4). Peak torque dropped by an average of 22% in all groups; stiffness only changed in the RF/ECC group (*p* = 0.04). Muscle work during the damage protocol was lower for AF/ECC than for ECC and RF/ECC (*p* = 0.005).

**Conclusion:**

Hamstring muscle damage was comparable between the three groups. However, the AF/ECC group resulted in the same amount of muscle damage while accumulating significantly less muscle work during the protocol of the damage exercise.

**Trial registration:**

This study was preregistered in the international trial registration platform (WHO; registration number: DRKS00025243).

**Supplementary Information:**

The online version contains supplementary material available at 10.1007/s00421-023-05234-z.

## Introduction

Muscle injuries are the most frequent injury type in soccer, eliciting enormous periods of downtime for players as well as financial costs for clubs (Ekstrand et al. 2009; Hägglund et al. 2013). Observations from the UEFA study by Ekstrand et al. (2009) revealed an average downtime of almost 37 days (~ 12%) per soccer season, with a sixfold increase of injury risk during matches compared to training. In 82–92% of all cases, muscle injuries affect the lower extremities, with the hamstrings commonly being referred to as the muscle group most susceptible to injury in professional soccer (Hägglund et al. 2013). Reaching up to 7 muscle strains per team per season (Ekstrand et al. 2009), hamstring injuries are highly associated with the intense, high-speed exertion profile in soccer (Askling et al. 2007), and routinely occur without opponent contact (Ekstrand et al. 2011). In addition to intrinsic risk factors such as age, gender, or injury history, muscular fatigue has been discussed as a potential contributor (Ekstrand et al. 2009; Hägglund et al. 2013), given that the number of hamstring injuries appear to increase towards the end of the matches’ halves. Both accumulated muscular fatigue and a decrease in the eccentric hamstring strength after the halftime interval might contribute to the increase in injury numbers (Ekstrand et al. 2011). Furthermore, a recent publication of Ekstrand et al. (2021) reported that an increasing number of matches played is associated with increased injury risk. A possible reason for this could be residual muscle fatigue (Hader et al. 2019). Residual fatigue has been reported to be a prolonged form of accumulated post-match fatigue, which is accompanied by fatigue-induced impairments of skeletal muscle, e.g., decreases in muscle force or raise in creatine kinase, and requires several days to fully recover from (Silva et al. 2018).

Exercise-induced muscle damage (EIMD) and its associated symptoms of delayed-onset muscle soreness (DOMS) have been assumed to represent a precursor of structural muscle injuries by the experts of the Munich consensus statement (Mueller-Wohlfahrt et al. 2013), and have been classified as a functional muscle injury (type 1B). While the authors stated that persistent, unrecognized DOMS could possibly cause further structural injuries, they also acknowledged that more research is necessary to investigate the exact implications of DOMS for increased injury risk. Hence, indirect muscle damage markers could indicate a risk of structural damage and allow researchers to investigate potential injury risk factors without risking an injury. These reports of the Munich consensus statement also indicated that muscle fatigue (in form of fatigue-induced muscle disorders), DOMS and more severe muscle injuries should not be accounted for as independent entities. A possible overlap between muscle fatigue and muscle injuries has also been considered in the excellent review by Allen et al. (2008).

Despite these findings and their potential implications for injury prevention in elite sports, only a handful of studies with the aim to address the influence of muscular fatigue on exercise-induced hamstring muscle damage have been conducted in an experimental research setting. In addition, these studies have yielded controversial results, creating ambiguity on the extent to which muscular fatigue influences EIMD and the risk of further structural damage. Nosaka and Clarkson (1997) reported preventive effects of previous concentric exercise on EIMD. The participants of their study performed 100 concentric biceps curls followed by 12 maximal eccentric biceps curls. The authors argued that the provoked fatigue may have decreased the participants’ force production during the eccentric exercise, and consequently, the mechanical load applied to the muscle was not sufficient for evoking damage. Contrary, Wilmes et al. (2021) showed that acutely fatigued hamstrings have large decreases in maximal voluntary contraction and electromyography activity after a simulated soccer match. Further, they found larger fatiguability in the long head of the biceps femoris than in the medial hamstrings. The authors concluded that muscle fatigue alters the running mechanics of the subjects, which ultimately increases hamstring injury risk. In accordance, two studies by Cohen et al. (2015) and Coratella et al. (2015) showed altered hamstring to quadriceps strength ratios after a simulated soccer match, with larger decreases in eccentric hamstring peak torque compared to quadriceps peak torque. Lastly, Gleeson et al. (2003) reported similar results. In their longitudinal study, the participants performed concentric biceps curls (60% and 70% of one repetition maximum) with their non-dominant arm over a period of four weeks, followed by an eccentric training protocol that was completed with the fatigued and the control arm. The fatigued arm resulted in greater force reduction, muscle soreness and circumference compared to the control arm, with the increased circumference interpreted as a sign for greater inflammation and muscle swelling. Further, they discussed that the fatiguing precondition stiffened contractile properties prior to the eccentric training regimen, thereby likely increasing susceptibility to overextension and damage (Gleeson et al. 2003).

Overall, the available research regarding the exact effects of muscle fatigue on EIMD is unclear. Further, it is currently difficult to draw conclusions on the extent of which acute and residual fatigue influence muscle damage in recreational or professional athletes, especially in the hamstring muscles. Given that in contact sports traumatic injuries are usually insurmountable to prevent, prevention programs and close athlete monitoring might offer options to counteract overuse hamstring injuries (Chebbi et al. 2020). To implement such prevention programs however, the exact influence of muscle fatigue on overuse injuries needs to be understood, given its proposed role as a central risk factor.

As the number of hamstring injuries rises towards the end of the matches’ halves, and with increased match frequency, the purpose of this study was to investigate the effect of acute (AF) and residual (RF) muscular fatigue on EIMD of the hamstring muscles. Based on the observations by Ekstrand et al. (2009) and the reported findings of Wilmes et al. (2021) and Gleeson et al. (2003), the following hypothesis was established: (1) acute or residually pre-fatigued hamstring musculature displays greater susceptibility to EIMD compared to an unfatigued control group.

## Methods

### Experimental design

The three-armed, randomized controlled trial investigated the effects of acute and residual muscle fatigue on exercise-induced hamstring muscle damage in 24 male participants. Given that muscle damage cannot be adequately assessed by a single parameter (Clarkson and Hubal 2002), we opted for a holistic, yet specific approach and investigated muscle responses by peak torque, sonography [muscle thickness], tensiomyography [TMG], shear wave elastography [SWE], range of motion [ROM], creatine kinase [CK], and pain perception (visual analogue scale [VAS]). All measurements and training interventions were conducted in our laboratory by the same investigators. The participants started with an initial measurement, consisting of an ultrasound examination (muscle thickness, SWE), a fatigue test (1 × 50 concentric leg curls), and an examination of the contractile properties (TMG) of the *m. biceps femoris* [BF]. The exact anatomical structures of the BF were examined through palpation and ultrasound diagnostics by an experienced investigator at the start of the study. Further, the correct positioning on the muscle was evaluated by SWE, which has been used as a valid method on the hamstring in various studies (Avrillon et al. 2020; Kawama et al. 2022; Nakamura et al. 2016; Voglar et al. 2022). Based on muscle stiffness, subjects were randomly (www.randomizer.org) assigned to one of three training groups (eccentric training group [ECC], *n* = 8; acute fatigue + eccentric training group [AF/ECC], *n* = 8; residual fatigue + eccentric training group [RF/ECC], *n* = 8). The study was approved by the local ethics committee and was conducted in accordance with the ethical standards set by the declaration of Helsinki. Further, the study was pre-registered at the German register for clinical trials (DRKS00025243).

### Participants

An a priori power analysis was conducted using G*Power 3.1 (University Duesseldorf, Germany). The analysis deemed that 24 participants are necessary for a power of 0.80, with an effect size of *f* = 0.2 and an *α* = 0.05, based on the effects previously reported (Nosaka and Clarkson 1997). Therefore, 24 male participants were recruited for this study. Baseline characteristics of the study population are presented in Table 1 and were assessed using a 3D body scanner (Scaneca GmbH, Berlin, Germany). Only male participants were included as sex has been reported to influence EIMD (Markus et al. 2021) and given that the aim of this study was to provide transferable results for male professional soccer players. Inclusion criteria required the participants to be between 18 and 35 years of age and to have an average training volume of at least two training sessions per week, which has been reported to represent the minimal dosage in order to maintain strength and aerobic performance (Spiering et al. 2021). Subjects who (1) were not between 18 and 35 years, (2) completed less than two exercise sessions per week, (3) regularly took medications, or, (4) suffered from acute lower extremity injuries were excluded from study participation.

**Table 1:** Baseline characteristics of study participants

	Age (y)	Height (cm)	Weight (kg)	Body Fat (%)	BMI (kg/m^2^)	FFMI (kg)
ECC	25.75 ± 2.54	185.63 ± 7.46	86.58 ± 7.35	16.19 ± 4.16	25.69 ± 1.67	20.99 ± 1.19
AF/ECC	28.25 ± 3.99	181.13 ± 5.08	81.06 ± 9.94	15.2 ± 6.07	24.85 ± 4.18	20.56 ± 2.31
RF/ECC	24.9 ± 2.64	179.5 ± 7.25	78.34 ± 6.64	13.95 ± 4.26	24.35 ± 2.16	20.95 ± 1.72

Data in means ± SD

No group differences were found for baseline characteristics

*ECC* eccentric training group, *AF/ECC* acute fatigue + eccentric training group, *RF/ECC* residual fatigue + eccentric training group, *BMI* body mass index, *FFMI* fat free mass index

The weekly hours of training were documented based on the participants’ estimates prior to the study (Table 2). During the study, participants were instructed to maintain their habitual training regimen. All participants were elucidated about the goal of the study as well as its conduction and provided informed written consent of participation.

**Table 2:** Habitual training regimen of study participants prior study start

	Resistance training per week (h)	Endurance training per week (h)	Competitive sports per week (h)	Total training per week (h)
ECC	4.75 ± 4.06	2.63 ± 1.60	1.25 ± 1.75	8.63 ± 3.07
AF/ECC	4.06 ± 3.88	1.38 ± 1.30	2.44 ± 3.20	7.88 ± 3.04
RF/ECC	2.25 ± 1.73	0.75 ± 0.85	5.25 ± 5.64	8.25 ± 4.87

Habitual training volume was documented based on participants’ estimates

Data in means ± SD

No group differences were exposed for habitual training volume per week

*ECC* = eccentric training group, *AF/ECC* = acute fatigue + eccentric training group, *RF/ECC* = residual fatigue + eccentric training group

### Muscle damage protocol

All groups performed an eccentric training session on an isokinetic dynamometer (ISOMED 2000, D. & R. Ferstl GmbH, Hemau, Germany), consisting of 6 × 10 eccentric leg curls with maximum effort, separated by a one-minute resting interval between sets as described by Brusco et al. (2018). Subjects were seated with an 85° trunk flexion; knee joint range of motion was set from a 90° flexed to a 10° extended position. Participants were tightly strapped to the ISOMED 2000 at the shoulders, hip, knees, and ankles to minimize body movement. Besides, the axis of rotation was aligned to the lateral femoral condyle, followed by gravitational moment correction (Zhang et al. 2021). As hamstring injuries in soccer occur mainly during explosive movements (Silva et al. 2018) and the goal of this study was to provide applicability to professional soccer, the angular velocity was set to 210° s^−1^ during the eccentric phase, which has been demonstrated to evoke muscular damage while approximating the contraction velocity in explosive sports such as soccer to a larger extent (Chapman et al. 2006). During the concentric phase of the movement, the leg was passively moved with an angular velocity of 50° s^−1^.

### Fatigue protocol

**Fig. 1:**
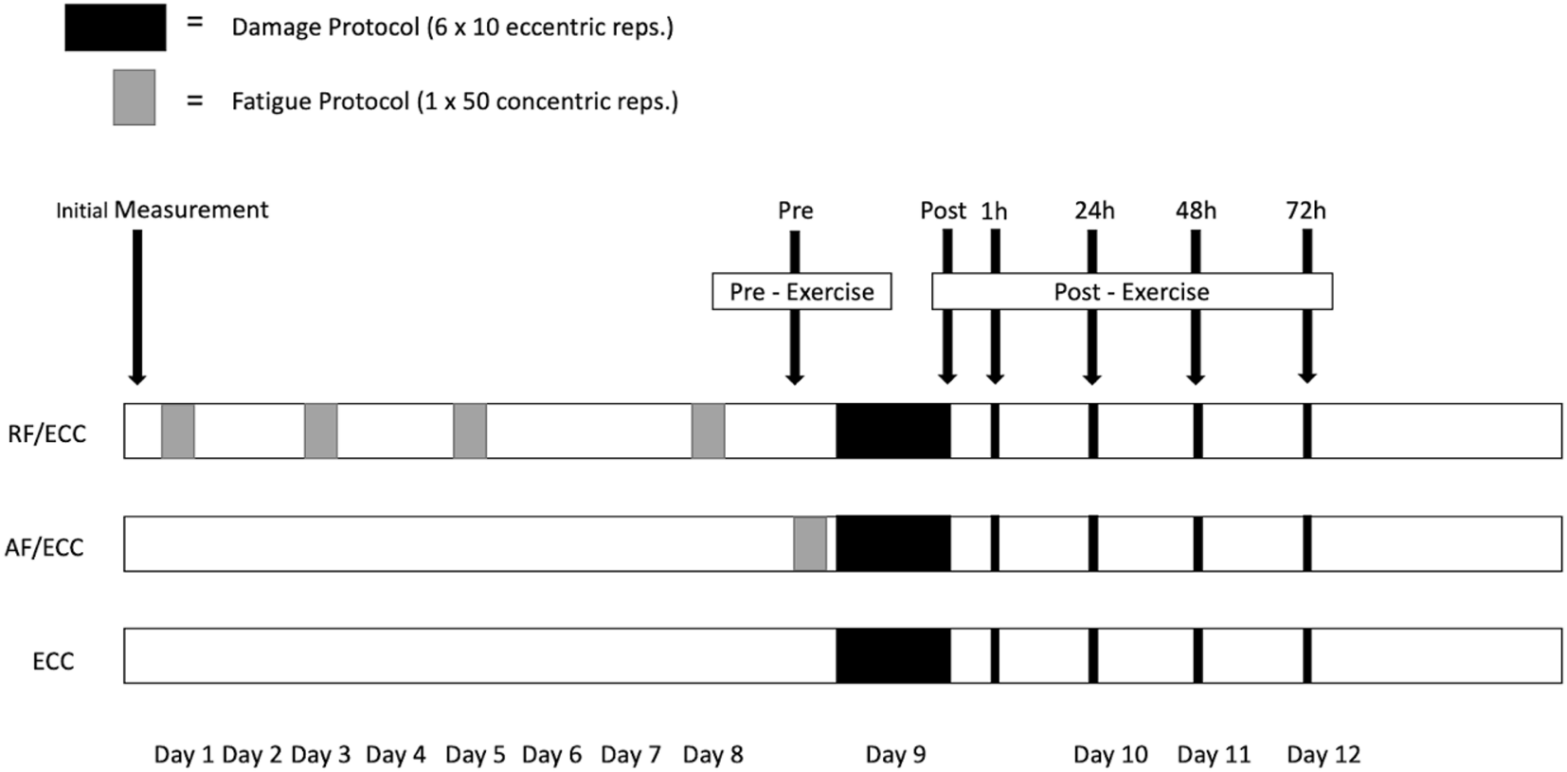
Graphical illustration of the study design. All groups started with an initial measurement. The damage protocol consisted of 6 × 10 eccentric hamstring curls and was performed by each group. Measurements were conducted pre, post, 1 h, 24 h, 48 h and 72 h post. While the AF/ECC group performed the fatigue protocol (1 × 50 concentric hamstring curls) immediately before the damage protocol, the RF/ECC group performed the same protocol on days 1, 3, 5, and 8. *ECC* eccentric training group, *AF/ECC* acute fatigue + eccentric training group, *RF/ECC* residual fatigue + eccentric training group

The fatigue training session consisted of 1 × 50 concentric leg curls with maximum effort (Costa et al. 2018). Angular velocity was set to 210° s^−1^ for the concentric phase of the movement and to 50° s^−1^ for the eccentric phase. The AF/ECC group performed the fatigue session only once, immediately prior to the damage session. As the number of hamstring injuries increases with ongoing match duration (Ekstrand et al. 2009; Hägglund et al. 2013), the intention for this group was to create a soccer-specific situation, e.g., after an intense sprint towards the end of the match. The RF/ECC group performed the fatigue training session four times within eight predefined days leading up to the damage protocol (Fig. 1). We particularly aimed to create a loading pattern that simulates an international week with several high intensity matches, as increased match frequency and duration between matches both influence injury incidence (Bengtsson et al. 2013).

### Study parameters

Study parameters were measured before the damage session (pre), directly after it (post), 1 h after it (1 h post), and on the consecutive three days (24 h, 48 h, and 72 h post). Times of assessment on consecutive days were kept constant to prevent influences of circadian rhythm. Muscle thickness, stiffness and contractility were assessed on the BF on two measurement sites (mid and distal), as previous research has indicated varying responses to muscle damage within a single muscle after eccentric exercise (Maeo et al. 2018).

### Peak torque and fatigue ratio

Peak torque assessment of the knee flexors has been shown to have excellent reproducibility (Dirnberger et al. 2012), with inter-day repeatability being excellent (ICC: 0.92 [CI 95% 0.80–0.96]). After a general five-minute warm-up on a bicycle ergometer, the testing regimen consisted of 2 × 5 maximal eccentric hamstring contractions at a pre-set angular velocity of 210° s^−1^, based on a study by Abdel-aziem et al. (2018). The two sets were preceded by a warm-up set, with the participants only using 80% of their self-estimated maximal effort. Between each set was a three-minute resting interval. To calculate the fatigue ratio during the fatigue session (1 × 50 concentric repetitions), the relationship of the performed work during the first third of the repetitions (first 16 repetitions) and the performed work of the last third of the repetitions (last 16 repetitions) was used (Saenz et al. 2010).

### Creatine kinase

80 μl of capillary blood were collected from the participants’ earlobe and immediately centrifuged (Universal 320 R, Andreas Hettich GmbH, Tufflingen, Germany) for 10 min at 3000 rounds per minute. After centrifugation, 10 μl of blood plasma was collected with a reusable pipette (Eppendorf Research Plus, Eppendorf, Hamburg, Germany), pipetted on a dry slide reagent, and analyzed in a point-of-care-testing system (DRI-CHEM Analyzer FDC NX500, Fujifilm Europe, Duesseldorf, Germany). Post-exercise samples were manually diluted in the form of a dilution series (1:2; 1:4; 1:8; 1:16; 1:32; 1:64), using a concentrated 0.9% sodium chloride solution. The lowest dilution was placed on the dry slide reagent for analysis, and if the value was not detectable, the next highest dilution was applied. Finally, the undiluted value was calculated by multiplying the diluted value with the dilution factor.

### Range of motion and pain perception

ROM was measured, using the 90/90 test for hamstring flexibility, with a double-armed, digital scale goniometer (Digital Angle Ruler, Preciva, China) placed on the inside of the popliteal fossa. Participants were lying in a supine position on an examination table and were told to raise their straightened, dominant leg as high as possible (Abdel-aziem et al. 2018). Perceived pain sensation was assessed using the VAS. The scale was exactly 100 mm long (3.94 inches) and ranged from “no pain at all” to “worst pain imaginable” (Heller et al. 2016). The participants were instructed about the proper utilization of the scale before each measurement, as described by Schwiete et al. (2021).

### Tensiomyography

**Fig. 2:**
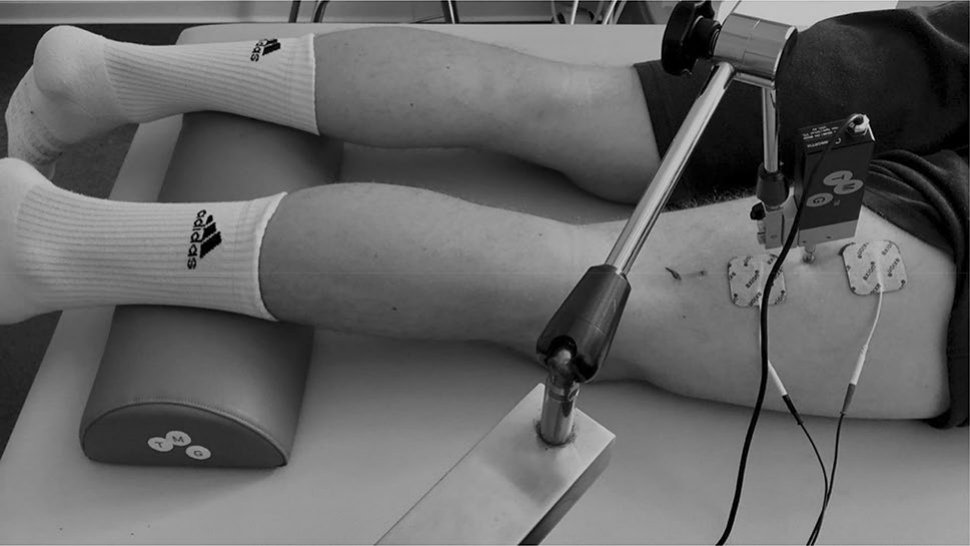
TMG set-up. Participants were lying in a prone position, with a TMG cushion placed under the lower leg to create a knee flexion of ~ 5°. Muscle contractility was assessed at two locations. BF-mid represents the measurement location at 50% of the BF length (ischial tuberosity to fibula head). BF-distal was exactly 5 cm distal of BF-mid

TMG measurements (TMG-BMC Ltd., Ljubljana, Slovenia) were conducted on the BF, with the participants lying in a prone position. Their ankles were placed on the TMG cushion for lower leg measurements in order to generate a knee flexion of about 5° (Wilson et al. 2019). We assessed muscle contractility at two measurement locations on the BF (Fig. 2). The first position was at 50% of the length between the origin (ischial tuberosity) and the insertion (head of fibula) (BF-mid). The second position was exactly 5 cm distal (BF-distal). Two electrodes (self-adhesive, dura-stick, 50 × 50 mm) with an inter-electrode distance of 5 cm were attached onto the prepared skin (Piqueras-Sanchiz et al. 2020). The location was marked with a water-resistant pen. To ensure that the electrodes were correctly placed on the muscle belly of the BF, each participant was instructed to perform an isometric hamstring muscle action at 90° knee flexion against the assessor’s arm. Starting at 50 mA, the intensity was incrementally increased by 10 mA every 30 s until either maximal deformation (D_m_) or maximal output (110 mA) was reached (Roth et al. 2021). Contraction velocity (V_c_) was calculated as the mean velocity until 90% D_m_ (V_c_ = 0.9 D_m_/[T_d_ + T_c_]*1000) was reached (Roth et al. 2021).

### Shear wave elastography

SWE is a form of sonography that allows the quantification of elastic and mechanical properties of tissues, e.g., muscle stiffness (Ryu and Jeong 2017), with previous research confirming good to excellent reliability of SWE (ICC: 0.82–0.92 [CI 95% 0.66–0.92]) (Kozinc and Šarabon 2020). Information regarding muscle stiffness after exercise may help researchers and practitioners broaden their understanding of muscle injuries and their respective healing processes (Ryu and Jeong 2017), as increased muscle stiffness is a reported symptom of fatigue-induced muscle disorders (injury type 1A) (Mueller-Wohlfahrt et al. 2013). An ACUSON Redwood (Siemens Healthineers, Erlangen, Germany) was used in shear wave elastography mode with a musculoskeletal preset to assess muscle stiffness. All measurements were conducted by the same experienced investigator. A 10L4 linear array probe (50 mm width) was placed longitudinally at BF-mid and BF-distal and adjusted to match the orientation of muscle fibers. Anatomical landmarks, imaged by the ultrasound system, were used as reference marks for the region of interest. Region of interest was 5 × 10 mm and was placed beneath the upper fascial sheath of the muscle. Within the region of interest, we evaluated elastography values in a measuring circle (10 mm diameter). All SWE values are given as Young’s Modulus (E). Young’s Modulus describes the resistance of the tissue to deformation against an uniaxial compression or tension (Ryu and Jeong 2017), and is calculated from shear wave speed in the transverse plane (*cT*) and the muscle mass density (ρ), which is assumed to be constant at 1000 kg⋅m^−3^ (Andonian et al. 2016): E = 3 ρ *cT* ^2^. Three consecutive measurements were performed at both measurement sites, and means were used for the statistical analysis.

### Sonography

Muscle thickness of the BF was assessed using the same ultrasound device; participants were positioned as reported for TMG measurements. A 10L4 linear array probe (50 mm width) was held with minimal pressure over the marked site. Muscle thickness was measured from the lower margin of the upper fascia to the upper margin of the lower fascia. The ultrasound images were saved at each timepoint and were used as a reference for the following measurement to increase between-day repeatability, which has been shown to be excellent in our laboratory (ICC: 0.96 [CI 95% 0.91–0.98]) (Schwiete et al. 2021). Three consecutive measurements were performed at both measurement sites; means were used for the statistical analysis.

### Statistical analysis

A general linear two-way mixed analysis of variance (ANOVA) with repeated measures (group [3] × time [6]) was conducted for each dependent variable using SPSS (SPSS version 24.0, Chicago, IL, USA). Multiple comparisons with Bonferroni correction and Tukey’s post hoc test were conducted whenever main effects for time and group revealed significance. In case of a significant group × time interaction, simple main effects were interpreted separately by using repeated measures ANOVA (time) and one-way ANOVA (group). ANCOVA was used if baseline data differed significantly between groups. Preceding ANOVA, all data were checked for outliers and normal distribution using studentized residuals and Shapiro Wilk’s test of normality. Furthermore, Mauchly’s test of sphericity was carried out to confirm homogeneity of variance. If sphericity was not given, the Greenhouse–Geisser correction was interpreted. Once the Box’s test exposed statistical significance, the group × time interaction was not interpreted; instead, simple main effects were run as described. A separate one-way ANOVA with post hoc comparisons (Tukey) was used to investigate group differences of performed workload of the damage protocol. When the assumption of normality was violated, Friedman test was used to test for intra-group differences between measures; the Kruskal–Wallis test was used to assess group differences. All tests were based on a 5% level of significance. All data are presented in means ± SD for parametric tests, and as median (IQR) for non-parametric tests, respectively.

## Results

### Overall response to muscle damage

**Fig. 3:**
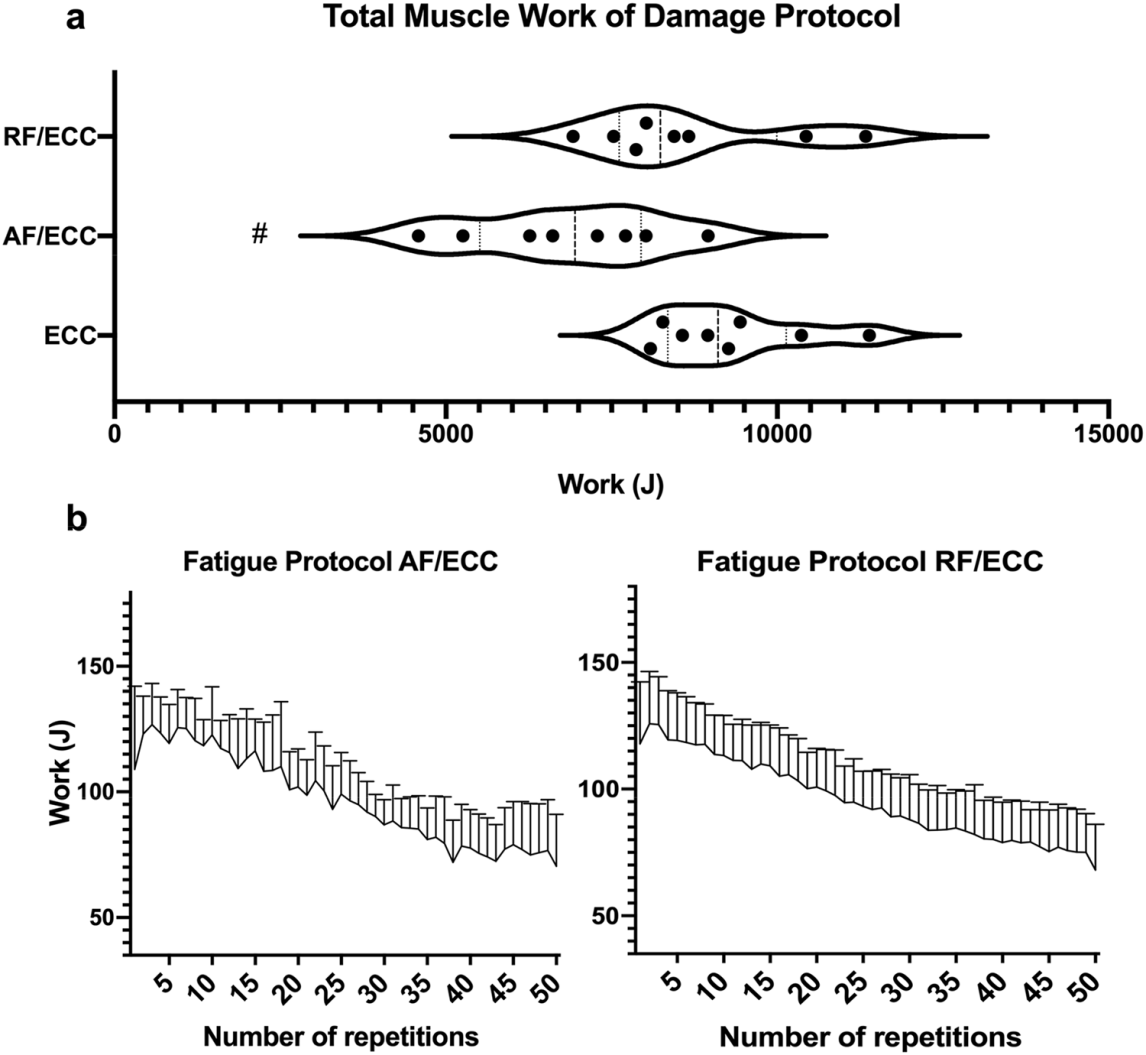
Changes in muscle work (J) during the muscle damage and the fatigue protocol of the hamstrings. **a** Shows total muscle work during the eccentric protocol for each group. Muscle work of the AF/ECC group was significantly lower than ECC and RF/ECC, as indicated by #; **b** Depicts the decrease in total muscle work over the course of 50 repetitions in the AF/ECC group and the RF/ECC group. *ECC* eccentric exercise group, *AF/ECC* acute fatigue + eccentric exercise, *RF/ECC* residual fatigue + eccentric exercise. In **a** the solid line within the violin plot represents the median; dashed lines show 1st and 3rd quartile. In **b** data are given as means ± SD

The accumulated muscle work of the damage training session (Fig. 3a) differed significantly between groups (*p* = 0.005). Eccentric muscle work of the AF/ECC group (6836.2 ± 1453.3 J) was significantly lower than that of the ECC group (9292.6 ± 1114.9 J, *p* = 0.005) and the RF/ECC group (8653.8 ± 1497.9 J, *p* = 0.04). There were no differences between the ECC group and the RF/ECC group (*p* = 0.6). During the fatigue session, the AF/ECC group amounted to a total of 6626.3 ± 727.1 J in muscle work, while decreasing by 31% throughout the training regimen. In comparison, muscle work of the RF/ECC group totaled to 4773.1 ± 749.4 J during the fatigue training and decreased by 33%. Muscle work of the RF/ECC group remained unchanged between the four fatigue sessions (*p* = 0.3) (Fig. 3b).

### Peak torque and range of motion

**Fig. 4:**
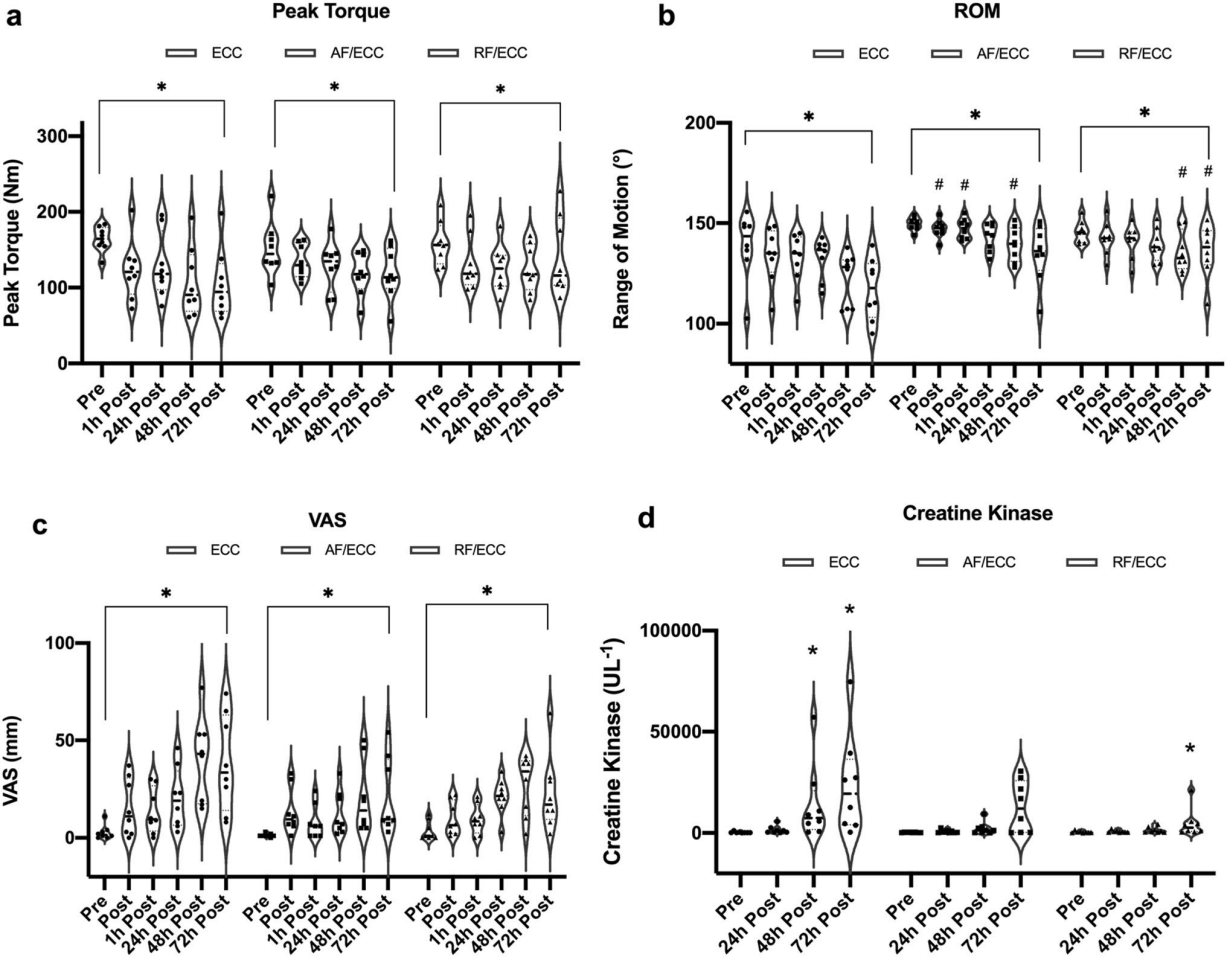
Changes in **a** peak torque, **b** range of motion, (ROM), **c** pain perception (VAS), and **d** creatine kinase after muscle damage. [*] = significant change over time/significant difference to baseline [#] = significant group difference to ECC. *ECC* eccentric training group, *AF/ECC* acute fatigue + eccentric exercise, *RF/ECC* residual fatigue + eccentric exercise. The solid line within the violin plot represents the median; dashed lines show 1st and 3rd quartile

There was no significant group × time interaction for peak torque (*p* = 0.1, η^2^ = 0.1). While main effects for groups revealed no statistical significance as well (*p* = 0.7, partial η^2^ = 0.2), main effects for time demonstrated a significant change over time (*p* < 0.001, partial η^2^ = 0.4) (Fig. 4a).

Given that the Box’s test exposed significance, the group × time interaction for knee joint range of motion was not interpreted. Simple main effects showed significant changes over time for the ECC (*p* = 0.005, partial η^2^ = 0.6), the AF/ECC (*p* = 0.02, partial η^2^ = 0.5), and the RF/ECC group (*p* = 0.02, partial η^2^ = 0.5). All three groups had a reduction in knee joint range of motion over time, with the ECC group decreasing from 138.9 ± 16.7° to 117.2 ± 15.4° (∆ − 21.8 ± 19.2°), the AF/ECC group from 149.6 ± 3.2° to 134.9 ± 14.5° (∆ − 14.8 ± 14.8°) and the RF/ECC group from 145.5 ± 5.5° to 136.2 ± 13.2° (∆ − 9.4 ± 11.4°) (Fig. 4b).

### Pain perception and creatine kinase

No significant group × time interaction was revealed for subjective pain perception (*p* = 0.2, partial η^2^ = 0.1). Main effects for groups were not significant as well (*p* = 0.2, partial η^2^ = 0.1). All three groups reported an increase in pain perception, peaking at 48 h (2.8 ± 3.6 mm vs. 40 ± 21.8 mm [ECC], 1.3 ± 0.9 mm vs. 20.3 ± 18.2 mm [AF/ECC], 3.6 ± 4.9 mm vs. 27 ± 15.5 mm [RF/ECC]) and decreasing again at 72 h post-exercise (Fig. 4c).

As the assumption of homogeneity of variance and normality of distribution were violated for CK values, Friedman test was used as a non-parametric alternative for repeated measures ANOVA. Accordingly, median, and interquartile range instead of mean values were considered for CK concentration. CK concentration increased significantly in the ECC group (*p* < 0.001), the AF/ECC group (*p* = 0.04), and the RF/ECC group (*p* = 0.008) over time ([pre vs. 72 h]: ECC: 170.5 UL^−1^ (interquartile range: 138.0–319.3 UL^−1^) vs. 19,352 UL^−1^ (interquartile range: 4070.0–36,364.0 UL^−1^), AF/ECC: 227.5 UL^−1^ (interquartile range: 133.5–414.3 UL^−1^) vs. 12,035 UL^−1^ (interquartile range: 230.0–25,888.0 UL^−1^), RF/ECC: 410.5 UL^−1^ (interquartile range: 176.3–650.0 UL^−1^) vs. 2560 UL^−1^ (interquartile range: 706.0–5459.3 UL^−1^). Post-hoc analysis revealed significant increases from pre in the ECC group at 48 h (∆ 13,804 UL^−1^, *p* = 0.003), and 72 h post-exercise (∆ 23,324 UL^−1^, *p* < 0.001). CK concentration in the RF/ECC group (∆ 4352 UL^−1^, *p* = 0.006) revealed a significant increase at 72 h post. Kruskal–Wallis test exposed no group differences for CK at any time point (all *p* > 0.05) (Fig. 4d).

### Tensiomyography

**Fig. 5:**
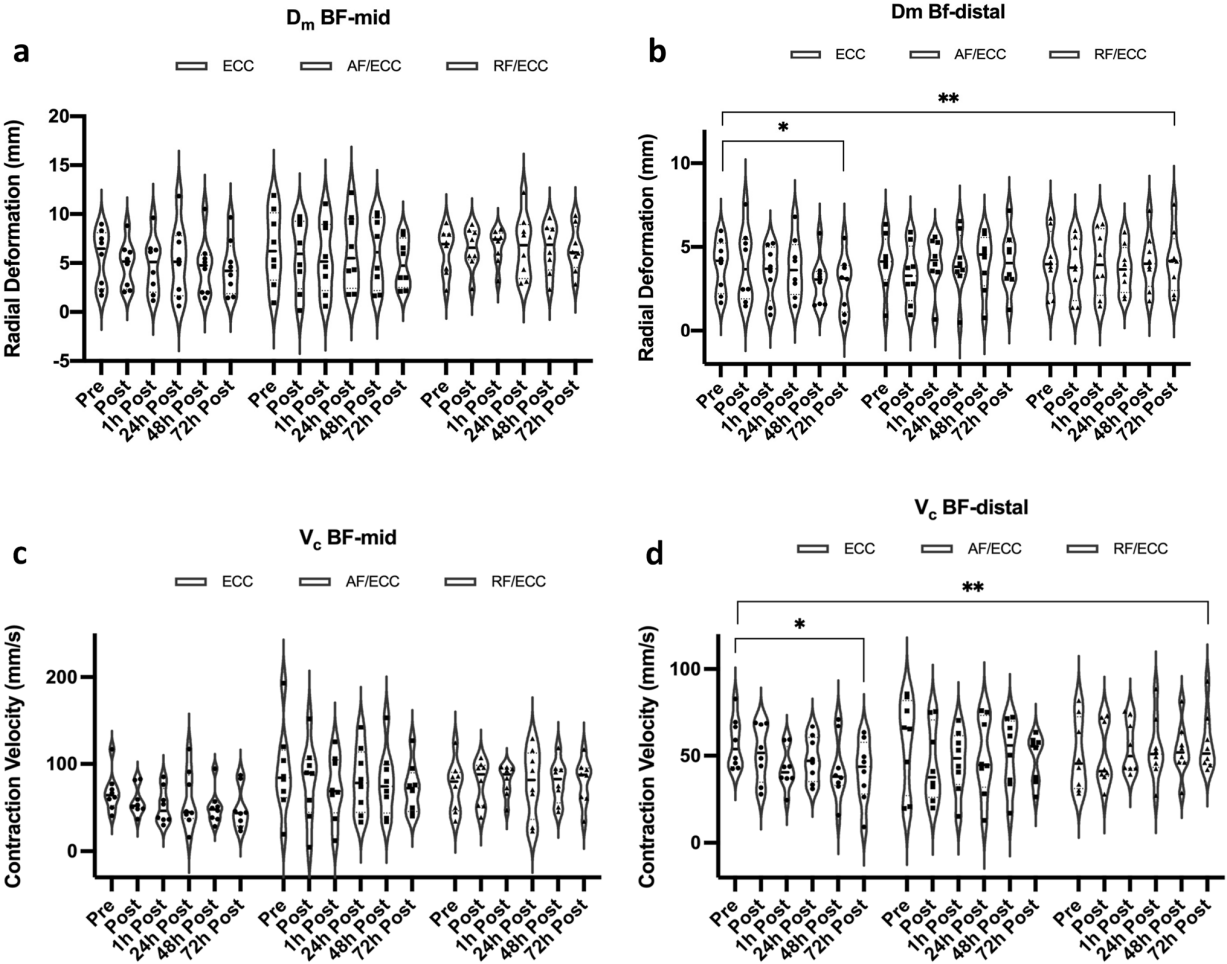
D_m_ and V_c_ at BF-mid (**a** + **c**) and BF-distal (**b** + **d**) after muscle damage. [*] = significant changes over time. [**] = significant group × time interaction. *BF-mid* 50% length of biceps femoris, *BF-distal* 5 cm distal of BF-mid, *ECC* eccentric training group, *AF/ECC* acute fatigue + eccentric exercise, *RF/ECC* residual fatigue + eccentric exercise. The solid line within the violin plot represents the median; dashed lines show 1st and 3rd quartile

There was no group × time interaction for D_m_ at BF-mid (*p* = 0.7, partial η^2^ = 0.06); no significant effects could be revealed for group (*p* = 0.5, partial η^2^ = 0.06) or time (*p* = 0.2, partial η^2^ = 0.07): pre to 72 h post-exercise ∆ − 1.03 ± 1.18 mm [ECC], ∆ − 1.54 ± 1.9 mm [AF/ECC], and ∆ 0.2 ± 2.6 mm [RF/ECC] (Fig. 5a).

Conversely, there was a significant group × time interaction for D_m_ at BF-distal (*p* = 0.01, partial η^2^ = 0.2). Simple main effects for group were not significant at any time point (all *p* > 0.05). D_m_ at BF-distal decreased from 3.9 ± 1.6 mm at pre to 2.8 ± 1.7 mm (∆ − 1.05 ± 1.17 mm) after 72 h in the ECC group, but not in the AF/ECC (∆ 0.1 ± 1.4 mm) and RF/ECC group (∆ 0.1 ± 0.5 mm), respectively (Fig. 5b).

There was no significant group × time interaction for V_c_ at BF-mid (*p* = 0.5, partial η^2^ = 0.08). Main effects for group (*p* = 0.2, partial η^2^ = 0.2) and time (*p* = 0.3, partial η^2^ = 0.1) were not significant either. While V_c_ at BF-mid decreased from baseline by ∆ − 19.3 ± 13.8 mm/s in the ECC group and by ∆ − 19.5 ± 29.1 mm/s in the AF/ECC group after 72 h, it increased by ∆ 6 ± 30.9 mm/s in the RF/ECC group (Fig. 5c). A significant group × time interaction for V_c_ was revealed at BF-distal (*p* = 0.01, partial η^2^ = 0.2). While simple main effects for group were not statistically significant at any time point, simple main effects for time revealed significant changes over time for the ECC group (*p* = 0.004, partial η^2^ = 0.4), with V_c_ decreasing by ∆ − 16.2 ± 15.2 mm/s from baseline to 72 h post. (Fig. 5d).

### Muscle stiffness

**Fig. 6:**
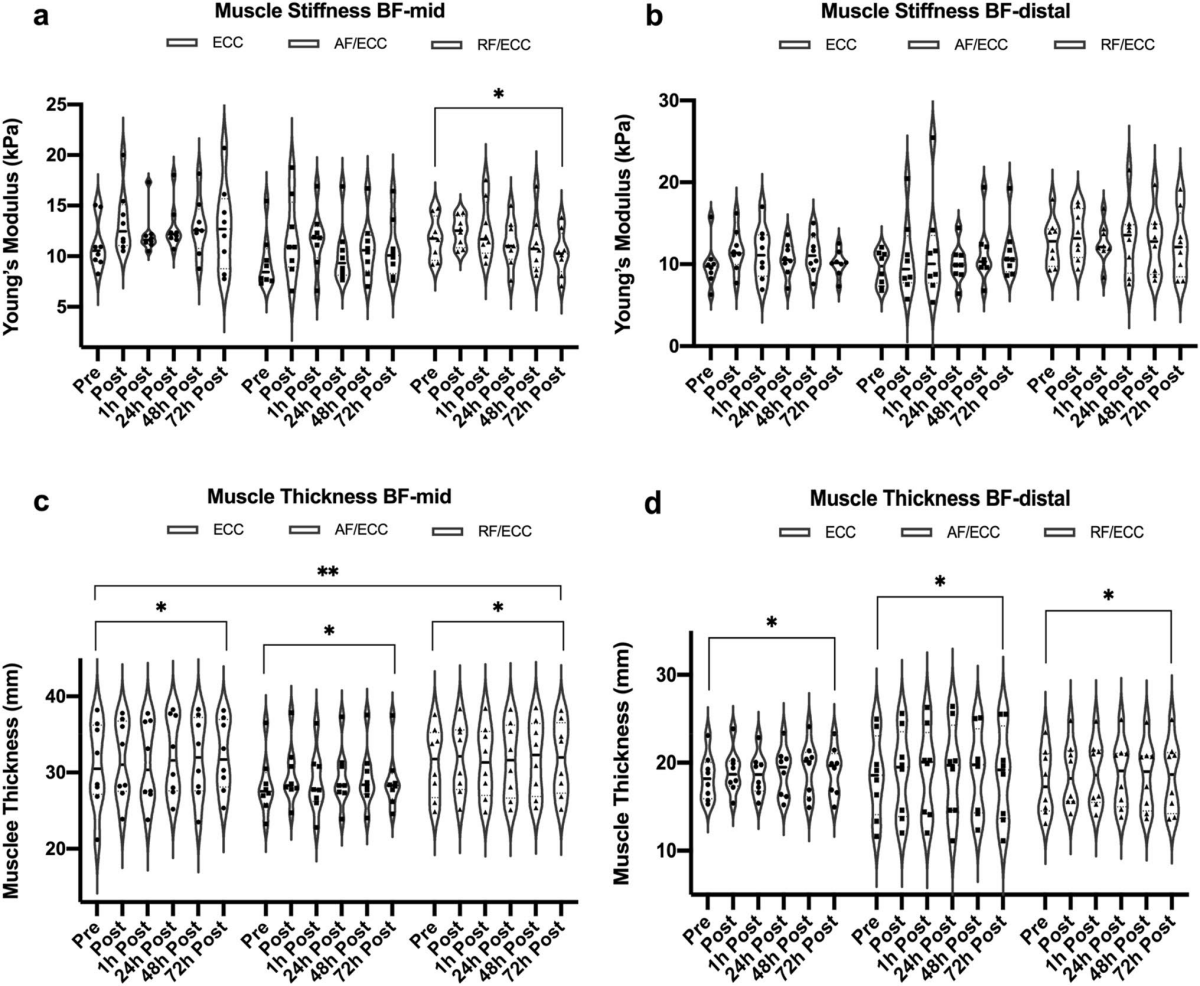
Changes in muscle stiffness and muscle thickness at BF-mid (**a** + **c**) and BF-distal (**b** + **d**) after muscle damage. [*] = significant changes over time. [**] = significant group × time interaction. *BF-mid* 50% length of biceps femoris, *BF-distal* 5 cm distal of BF-mid, *ECC* eccentric training group, *AF/ECC* acute fatigue + eccentric exercise, *RF/ECC* residual fatigue and eccentric exercise. Data in means ± SD

Mean acquisition depth for elastography was 1.75 ± 0.41 cm at BF-mid and 1.45 ± 0.31 cm at BF-distal. The Box’s test revealed significant differences for shear wave elastography at both BF-mid and BF-distal. Simple main effects of time revealed significant differences at BF-mid for the RF/ECC (*p* = 0.04, partial η^2^ = 0.3), but not for the ECC (*p* = 0.4, partial η^2^ = 0.1) or the AF/ECC group (*p* = 0.4, partial η^2^ = 0.1). Muscle stiffness at BF-mid increased in all groups from pre to post (ECC: ∆ 2.3 ± 2.7 kPa; AF/ECC: ∆ 3.6 ± 7.4 kPa; RF/ECC: ∆ 0.7 ± 1.5 kPa), staying elevated in the ECC and AF/ECC group ([pre vs. 72 h] ECC: 11.2 ± 2.5 kPa vs. 12.9 ± 4.3 kPa; AF/ECC: 9.5 ± 2.7 kPa vs. 10.7 ± 3 kPa) and decreasing in the RF/ECC group ([pre vs. 72 h] RF/ECC: 11.8 ± 2.1 kPa vs. 10.3 ± 2.2 kPa) after 72 h (Fig. 6a). No between-group differences were found. Conversely, simple main effects for time at BF-distal did not reveal any significant differences (ECC; *p* = 0.2, partial η^2^ = 0.2; AF/ECC; *p* = 0.3, partial η^2^ = 0.2; RF/ECC; *p* = 0.7, partial η^2^ = 0.1). While returning to baseline values in the ECC and RF/ECC group ([pre vs. 72 h] ECC: 9.9 ± 2.7 kPa vs. 10.1 ± 1.6 kPa; RF/ECC: 12.7 ± 2.9 vs. 12.5 ± 4.1 kPa), muscle stiffness stayed elevated in the AF/ECC group ([pre vs. 72 h] AF/ECC: 9.5 ± 2.1 kPa vs. 11.5 ± 3.4 kPa) (Fig. 6b). Given that muscle stiffness differed at baseline, ANCOVA, covarying for baseline data, was used to assess between-group differences for each measurement; however, ANCOVA did not reveal significance either.

### Muscle thickness

There was a significant group × time interaction for muscle thickness at BF-mid (*p* = 0.02, partial η^2^ = 0.2). Simple main effects for time revealed significant changes for the ECC (*p* = 0.01, partial η^2^ = 0.5), the AF/ECC (*p* = 0.02, partial η^2^ = 0.4), and the RF/ECC group (*p* = 0.04, partial η^2^ = 0.3). Muscle thickness at BF-mid increased slightly from pre to post (ECC: ∆ 0.6 ± 1.0 mm; AF/ECC: ∆ 1.4 ± 1.1 mm; RF/ECC: ∆ 0.6 ± 0.7 mm), remaining elevated after 72 h ([pre vs. 72 h] ECC: 30.9 ± 9.8 mm vs. 32.2 ± 4.7 mm; AF/ECC: 28.3 ± 3.9 mm vs. 28.9 ± 3.9 mm RF/ECC: 31.3 ± 4.6 vs. 31.8 ± 4.9 mm) (Fig. 6c). No between-group differences could be revealed for muscle thickness at BF-mid across measuring points. No significant group × time interaction could be shown for muscle thickness at the BF-distal measurement point (*p* = 0.5, partial η^2^ = 0.08). Main effects for time revealed statistical significance (*p* = 0.008, partial η^2^ = 0.2; [pre to 24 h] *p* = 0.04), whereas main effects for groups did not (*p* = 0.9, partial η^2^ = 0.002). Muscle thickness at BF-distal increased from baseline by ∆ 0.7 ± 1.0 mm (18.3 ± 2.6 mm to 19.1 ± 2.7 mm) in the ECC group, by ∆ 0.1 ± 1.4 mm (18.4 ± 4.7 mm to 18.6 ± 5.3 mm) in the AF/ECC, and by ∆ 0.6 ± 1.0 mm (17.8 ± 3.7 mm to 18.4 ± 4.1 mm) in the RF/ECC group after 72 h (Fig. 6d).

## Discussion

The main aim of this study was to investigate the effects of AF and RF on EIMD in hamstring muscles and thus, to draw possible conclusions on how muscle fatigue influences repetitive muscle damage during a season, which could possibly lead to overuse injuries. Collectively, neither AF nor RF affected the extent of EIMD in the hamstring muscles in this study population. However, the participants of the AF group showed similar amounts of muscle damage, while accumulating significantly less muscle work during the damage training session. Accordingly, in this study, less muscle work was needed to elicit the same amount of muscle damage in the acute fatigue group.

### Overall responses to muscle damage

As nearly all dependent variables (except D_m_ and V_c_ for BF-mid and SWE for BF-distal) showed significant time effects, the training regimen was successful in inducing muscle damage in all three groups. Contrary to our established hypothesis, neither the acute nor the residual fatigue group differed significantly compared to the non-fatigued group, as assessed by indirect muscle damage markers. Given that the number of hamstring injuries increases during the end of the matches’ halves and with increased match frequency, we expected the fatiguing preconditions to increase the susceptibility to EIMD, and therefore, to give a possible explanation for the elevated injury risk.

The AF/ECC group amounted significantly less total work (6836.2 ± 1.453.3 J) during their damage session than the ECC (9292.6 ± 1114.9 J, *p* = 0.005) and the RF/ECC group (8653.8 ± 1497.9 J, *p* = 0.04). Furthermore, their total work during the fatigue session decreased by over 30%. Collectively, this indicates that the acutely fatigued hamstrings result in the same amount of muscle damage while performing less work compared to the ECC and RF/ECC group. To the best knowledge of the authors, it should be noted that the relationship between the amount of muscle work and the resulting muscle damage is not fully understood. Changes of damage markers are influenced by several factor, including the contraction type, the recruited muscle fibers and the individual’s training level (Chapman et al. 2008). Even when controlling for these variables, there is still a large between-subject variation in post-exercise marker changes. While the training volume and contraction type was not significantly different between the AF/ECC and the ECC group, we have no information regarding other factors, e.g., fiber recruitment during the exercise session. Accordingly, our results should be interpreted with a degree of caution.

The current findings also deliver a possible explanation for the increased injury risk towards the end of soccer matches’ halves: the pre-fatigued muscle may not be able to accumulate the same amount of eccentric muscle work as the unfatigued hamstrings and thus, less muscle work resulted in the same amount of muscle damage. Especially in the early stages of the stretch, where the unfatigued muscle might still be able to mitigate an eventual strain, this inability to absorb energy may increase the injury risk (Mair et al. 1996). In a very recent publication by Ekstrand et al. (2023), they reinforced muscular fatigue as a potential risk factor for injuries. The findings of our study indicate that acute fatigue alters the muscular performance during eccentric exercise protocols, as the AF/ECC group accumulated less muscle work compared to the ECC group (*p* = 0.005) during their damage training. Understanding how muscle fatigue affects EIMD and possible subsequent structural damage is of great importance for coaches and physicians, as it is a modifiable factor that can be controlled through individual programing of training.

The participants of the RF/ECC group showed no signs of increased vulnerability to muscle damage. One possible explanation might be that the fatigue session intentionally had no components of eccentric load to mitigate the repeated bout effect leading up to the damage protocol. Eccentric loading during the fatigue sessions could have prohibited conclusions about whether the differences in muscle damage are the result of the repeated bout effect or the residual fatigue. However, in regards to the observations by Ekstrand et al. (2011), the increased injury risk might not solely be the result of concentric fatigue, but actually demonstrate the accumulation of concentric and eccentric fatigue, which requires longer periods of recovery than concentric fatigue and is commonly referred to as “prolonged low-frequency force depression” (Allen et al. 2008). Since soccer requires a high degree of eccentric strength, future fatigue protocols, aiming to elicit residual fatigue, should incorporate eccentric strength components. Although this would include the aforementioned problems of the repeated bout effect, it probably provides a better representation of the performance profile of soccer. It should be noted that the results of the RF/ECC should be interpreted with a certain degree of circumspection as we did not perform a direct assessment of residual fatigue before the muscle damage training. However, the fatigue protocol of the present study has elicited muscle fatigue in previous research (Costa et al. 2018) and evoked significant changes in acute fatigue in our study. Based on the acute changes, we assumed that residual fatigue was established throughout the intervention. While there were first signs of residual fatigue, the purely concentric training was either not exhausting enough to elicit residual fatigue, or the number of participants was too small to reveal significant effects. The absence of a direct assessment of residual fatigue might also be a reason why the RF/ECC group showed no significant differences compared to the other two groups.

### Peak torque and range of motion

All three groups reported significant reductions of eccentric peak torque following the damage session (*p* < 0.001). While the ECC group decreased by ∆ − 58.7 ± 36.9 Nm in peak torque, the AF/ECC and the RF/ECC group revealed decreases of ∆ − 33.4 ± 45.8 Nm and ∆ − 17.6 ± 40.9 Nm after 72 h, respectively. Loss in strength and power is a well-established symptom of DOMS (Cheung et al. 2003). Contrary to our expectations, no group differences were revealed for peak torque, indicating that neither AF nor RF significantly altered the loss of strength post-exercise. The current findings of the RF/ECC group are at odds with previous research from Gleeson et al. (2003), who reported that a four-week concentric training regimen resulted in larger reductions of strength after eccentric exercise, when compared to a control group. However, the results provide only limited comparability, as the authors assessed isometric peak torque, and their participants did not perform regular resistance training before the study started. Nosaka and Clarkson (1997) found less strength loss after concentric preconditioning of the elbow flexors and concluded that the concentric exercise may have a preventive effect on the muscle, as it possibly serves as a muscle warm-up and prepares the muscle for eccentric training. Based on the results of this investigation, the outcomes of Nosaka and Clarkson (1997) can only partly be confirmed. While the participants in our study also showed reduced muscle work during the damage session, both fatigue groups had a 30% decrease in total work during the preceding fatigue session. The amount of muscle damage was similar to the control group. Accordingly, the acute preconditioning fatigued the hamstrings before the muscle damaging session. This discrepancy between our study and the work of Nosaka and Clarkson (1997) could be explained by two factors: first, the lower extremity is subjected to more stress than the upper extremity during regular daily activities and thus could have a higher load capacity (Zhang et al. 2021). Second, while the subjects of Nosaka and Clarkson’s (1997) study did not participate in resistance training on a regular basis, our participants completed an average of 3.7 ± 3.4 h of strength training on a weekly basis. Accordingly, their performance level in the beginning of the workout was probably higher than that of an untrained individual due to a higher level of muscle recruitment and rate coding, thereby recording a larger loss of power. Collectively, the findings of this investigation contradict the idea that concentric preconditioning prevents muscle damage by serving as an activation. Rather, the fatiguing precondition weakened the muscle to a point where eccentric peak torque decreased, while less total muscle work was necessary to elicit these losses.

Interestingly, our results indicate that the fatiguing preconditioning limited the loss in knee ROM. While limited hamstring flexibility is not necessarily an injury indicator, its possible effects on muscle stiffness and other injury related components have been emphasized by Sanz et al. (2020). While the ECC group decreased by ∆ − 21.8° ± 19.2°, the AF/ECC and the RF/ECC group reported a loss of knee joint ROM by ∆ − 14.8° ± 14.8° and ∆ − 9.4° ± 11.4° after 72 h, respectively. Decreases in joint ROM after repetitive, eccentric exercise are a well-known symptom of DOMS (Cheung et al. 2003) and are associated with the swelling of connective and muscle tissue as part of an inflammatory response to muscle damage. Interestingly, previous research suggests varying effects of a soccer match on hamstring flexibility outcomes. While a recent study reported increased hamstring flexibility of professional players after a soccer match during the second half of a season (Kakavas et al. 2020), hamstring flexibility after a match is generally considered to be limited (Wollin et al. 2017). In their study, knee joint ROM was reduced until 72 h post-exercise, similar to the findings of this investigation.

### Creatine kinase and pain perception

All three groups revealed significant increases in CK values after the training protocols, with the study protocol evoking extremely pronounced CK values of up to 74.000 UL^−1^. Considering that trained populations usually show lower CK responses than untrained subjects (Brancaccio et al. 2008), the increases in our study underline the degree of muscle damage induced by the damage protocol.

The results of this investigation seem to confirm two theories regarding CK: first, the results indicate that CK concentration had not peaked at 72 h post-exercise, which is consistent with research reporting peak CK concentrations after 96 h (Brancaccio et al. 2008; Koch et al. 2014). Second, even though the ECC group had the largest increase in CK (∆ 23.324 UL^−1^, *p* < 0.001), the current results point towards the differentiation of high and low responders in terms of CK accumulation after muscle damage (Koch et al. 2014). Regardless of group allocation and the associated training protocols, participants showed extreme variance in CK values after 72 h (Fig. 4d). While the CK values of the RF/ECC group ranged from 512 to 21.232 UL^−1^, the differences within the AF/ECC (152 to 30.528 UL^−1^) and the ECC group (424 to 74.688 UL^−1^) were even more pronounced. This shows that CK varies widely between trained individuals that perform the same training regimen. One of the main factors affecting CK values after muscle damage is presumed to be the individual polymorphism of the ACTN3 gene (Koch et al. 2014), which was recently confirmed by de Lima et al. (2021). The results of their study showed that the CK response after muscle damage is attributed to the ACTN3 polymorphism, rather than load exposure itself. Therefore, muscle fatigue seems to play a rather minor role in the extent of CK accumulation after muscle damage.

Pain perception increased in all groups after the eccentric exercise (*p* < 0.001), showing typical symptoms of DOMS (Cheung et al. 2003). On average, pain perception peaked after 48 h, before starting to decrease again at 72 h post-exercise. Concentric preconditioning did not affect the magnitude of pain perception at any time point when compared to the unfatigued control group.

### Muscle thickness, muscle stiffness and muscle contractility

In all three groups, muscle thickness changed similarly after EIMD, both at BF-mid (*p* < 0.001) and BF-distal (*p* = 0.008) after 72 h. Peripheral muscle fatigue did not seem to influence muscle swelling in comparison to the unfatigued control group. An increase in muscle thickness, as a result of swelling after damaging exercise, is attributed to the inflammatory responses after EIMD (Vincent et al. 2010). Interestingly, intense metabolic conditions that occur during muscular fatigue might also trigger inflammatory responses that ultimately result in a swelling of the muscle cell (Behringer et al. 2014). Since there were no group differences in swelling in this study, muscular fatigue and damage do not appear to act in an additive manner on inflammatory responses, but in a rather synergistic pattern.

To extend the current methods, muscle stiffness and muscle contractility were assessed to examine changes after EIMD. To the authors’ knowledge, this was the first investigation to use SWE after heavy, eccentric exercise of the hamstring muscle and in a resistance-trained population. Most studies implemented fewer measurement occasions and merely investigated muscle stiffness in untrained populations (Lacourpaille et al. 2017; Leung et al. 2017). While there was no significant interaction for SWE, main effects of time revealed significance at BF-mid (*p* = 0.04). Accordingly, SWE exposed changes in muscle stiffness after a muscle damaging exercise regimen. Pairwise comparisons did not reveal significant changes between time points due to study power, and thus, only limited conclusions on the tendencies of muscle stiffness after eccentric loading can be derived. Overall, the literature provides contradictory data regarding SWE tendencies after eccentric loading. While several authors reported slight to large increases in muscle stiffness (Lacourpaille et al. 2017; Leung et al. 2017) after exercise, Xu et al. (2019) showed that muscle stiffness varies between muscle heads after training. In their study, muscle stiffness after eccentric training increased in the *m. rectus femoris*, slightly decreased in the *m. vastus lateralis,* and remained unchanged in the *m. vastus medialis oblique*. Hence, the authors concluded that muscle stiffness varies greatly between muscle heads after muscle damage, which might deliver a possible explanation for the absence of changes in muscle stiffness at BF-distal in our investigation. As we did not assess stiffness in the other hamstring muscles, i.e., *m. semimembranosus* and *m. semitendinosus*, no conclusion can be drawn. Given that several subjects also reported severe muscle soreness in the adductor muscles however, this hints to the possibility that the more medial muscle heads of the hamstrings were involved to a larger extent. Therefore, to evaluate stiffness, future research should include all hamstring muscles after eccentric training protocols. Similar to our findings, Kisilewicz et al. (2020) reported that muscle stiffness after EIMD varies within the respective muscle head, assessing SWE values at four measurement points on the *m. trapezius* (pars descendens) after 5 × 10 maximal eccentric contractions. Stiffness varied greatly at the measurement points, indicating spatial heterogeneity within the same muscle. This was confirmed by our results as SWE changed significantly in the RF/ECC group at BF-mid (*p* = 0.04), but not at BF-distal (*p* = 0.1).

In accordance with previous research, muscle contractility was hampered by muscle damaging exercise. Similar to the SWE values and confirming the results of Maeo et al. (2018), muscle contractility showed varying responses within a single muscle head after eccentric exercise. Significant group × time interactions were only revealed for BF-distal (D_m_: *p* = 0.01; V_c_: *p* = 0.01), but not for BF-mid. Further, only the ECC group revealed significant time effects for the TMG parameters D_m_ (*p* = 0.002) and V_c_ (*p* = 0.004), indicating that muscle contractility did not fully recover. In combination with the reported decreases in eccentric PT, these results confirm the idea of a post-contractile depression, which has been introduced by Allen et al. (2008). Post-contractile depression describes the prolonged force reduction that mainly occurs after low frequency stimulation, probably caused by reductions in sarcoplasmic reticulum Ca^2+^ release and Ca^2+^ sensitivity. The findings presented herein also extend the study by Harmsen et al. (2018), in which was proven that TMG serves as a suitable assessment method to detect muscle impairment after EIMD, even in a resistance-trained population. A possible explanation for this could be the reduced total muscular work during EIMD in the AF/ECC group. Due to the reduced work, fewer mechanical forces were exerted on the hamstring muscles, and the contractile units were stressed to a lesser extent. Further, a recent article, reviewing novel assessment methods to detect peripheral muscle fatigue, suggested that muscle fatigue results in a possible shift of load sharing within a group of synergistic muscle groups (Cè et al. 2020). The pre-fatigue of the AF/ECC and RF/ECC group could therefore have resulted in such a load sharing within the thigh muscles in order to prevent overloading of the hamstring muscles. Our findings show that changes after muscle damage in muscle contractility, muscle stiffness, and muscle thickness can vary greatly within muscle groups and even within the same muscle head. Accordingly, future researchers and practitioners should aim to collect these parameters at several measurement sites of a single muscle.

### Limitations

This study was not free of limitations. It should be noted that the results of the present study are based on indirect damage markers, which are associated with increased vulnerability to muscle damage, but do not optimally reflect the actual impact muscle fatigue might have on muscle injuries, i.e., fiber tears. A major limitation of this study is that we did not assess residual fatigue prior to the damage protocol. Further, the RF/ECC group had already completed the fatigue protocols before their pretest. This was done for the following reasons: First, we wanted to achieve an equal distribution of measurement time points between all groups and ensure the same number of measurements. Second, we wanted to investigate to what extent the fatigue training changed the baseline conditions for the RF/ECC group and thus, possibly led to an altered response to EIMD. Concentric-only residual fatigue did not influence EIMD in this study and is likely related to the fact that we did not include eccentric components to mitigate the repeated bout effect. In addition, we did not assess whether the participants of this study took nutrition supplements that might impact the inflammatory response to EIMD and subsequent performance measures. Future studies should aim to investigate the influence of different fatigue protocols to gain a better understanding on how residual fatigue might affect EIMD. Furthermore, this study had strict inclusion criteria, making the results difficult to apply to other populations, e.g., women.

## Conclusion

Overall, neither AF nor RF affected the extent of EIMD of the hamstrings. However, the participants of the AF/ECC group showed comparable muscle damage even though less muscle work was needed to elicit these effects. Further, muscle fatigue seemed to affect ROM and muscle contractility after muscle damage. Future studies should aim to investigate the influence of different fatigue protocols in order to gain a better understanding on how residual fatigue might affect EIMD. Lastly, the present study further confirmed that the novel muscle measurement methods of TMG and SWE can detect changes in muscle characteristics after EIMD and might serve as useful tools for team physicians and other practitioners to assess muscle characteristics.

### Perspective

The findings of the present study confirm the idea of previous research (Cohen et al. 2015; Coratella et al. 2015; Wilmes et al. 2021) that acutely fatigued hamstrings show increased susceptibility for EIMD, probably through an attenuated ability to absorb energy throughout eccentric contractions. Through the variety of assessed damage markers in this study, our results might help researchers and practitioners in the sports medical field to gain a better understanding of possible indirect damage markers for individual players. Muscle fatigue should be viewed as an integrative component in a complex system of factors affecting hamstring injury risk, that needs to be monitored acutely and chronically.

### Supplementary Information

Below is the link to the electronic supplementary material.

Supplementary file1 (DOCX 21 KB)

## Data Availability

The raw data supporting the conclusions of this article will be made available by the authors, without undue reservation.

## Abbreviations

AF: Acute fatigue
RF: Residual fatigue
AF/ECC: Acute fatigue + eccentric exercise
RF/ECC: Residual fatigue + eccentric exercise
ECC: Eccentric exercise
D_m_: Maximal displacement
V_c_: Contraction velocity
EIMD: Exercise-induced muscle damage
DOMS: Delayed-onset muscle soreness
TMG: Tensiomyography
SWE: Shear wave elastography
ROM: Range of motion
CK: Creatine Kinase
VAS: Visual analogue scale
BF: M. biceps femoris
ANOVA: Analysis of variance
ACTN3: Alpha-actinin-3 protein
