# Supplementary material for: Effects of muscle fatigue on exercise-induced hamstring muscle damage: a three-armed randomized controlled trial
Source: Eur J Appl Physiol. 2023 Jun 18;123(11):2545–61. doi: 10.1007/s00421-023-05234-z (PMC10616225; doi:10.1007/s00421-023-05234-z)
Supplement: Supplementary file 1 — Supplementary file1 (DOCX 21 KB) [file 421_2023_5234_MOESM1_ESM.docx]

**Supplementary Material**

**Table 3.** Representation of the changes of the investigated TMG parameters for both measurement points.

|  |  | **Pre** | **Post** | **1h Post** | **24h Post** | **48h Post** | **72h Post** |
| --- | --- | --- | --- | --- | --- | --- | --- |
| **D_m_ BF-mid (mm)** | ***ECC*** | 5.6 ± 2.9 | 4.8 ± 2.4 | 4.8 ± 2.9 | 5.2 ± 3.8 | 4.6 ± 3.0 | 4.5 ± 2.8 |
|  | ***AF/ECC*** | 6.5 ± 3.8 | 5.8 ± 3.6 | 5.6 ± 3.7 | 6.2 ± 3.8 | 6.0 ± 3.6 | 5.0 ± 2.5 |
|  | ***CF/ECC*** | 6.2 ± 2.4 | 6.5 ± 2.2 | 6.7 ± 1.8 | 6.7 ± 3.2 | 6.5 ± 2.5 | 6.4 ± 2.4 |
| **V_c_ BF-mid (mm/ms)** | ***ECC*** | 67.9 ± 22.8 | 57.7 ± 16.3 | 51.9 ± 20.3 | 58.5 ± 33.3 | 50.3 ± 20.1 | 48.7 ± 24.0 |
|  | ***AF/ECC*** | 91.5 ± 51.0 | 80.0 ± 45.1 | 73.0 ± 37.2 | 80.9 ± 38.0 | 78.9 ± 38.5 | 72.0 ± 28.9 |
|  | ***CF/ECC*** | 73.9 ± 29.5 | 77.7 ± 26.2 | 81.0 ± 17.9 | 78.4 ± 39.1 | 79.7 ± 24.4 | 79.9 ± 25.9 |
| **D_m_ BF-distal (mm)** | ***ECC*** | 3.9 ± 1.6 | 3.9 ± 2.2 | 3.5 ± 1.6 | 3.8 ± 1.8 | 2.9 ± 1.5 | 2.8 ± 1.7 |
|  | ***AF/ECC*** | 4.0 ± 1.7 | 3.4 ± 1.7 | 4.0 ± 1.6 | 4.0 ± 1.9 | 4.0 ± 1.8 | 4.1 ± 1.8 |
|  | ***CF/ECC*** | 4.1 ± 1.8 | 3.7 ± 1.8 | 4.1 ± 1.9 | 3.7 ± 1.4 | 4.1 ± 1.7 | 4.2 ± 1.9 |
| **V_c_ BF-distal (mm/ms)** | ***ECC*** | 57.2 ± 14.7 | 51.6 ± 16.5 | 42.7 ± 11.7 | 48.1 ± 13.1 | 42.5 ± 18.3 | 41.1 ± 18.1 |
|  | ***AF/ECC*** | 58.0 ± 26.4 | 45.0 ± 21.9 | 46.9 ± 18.0 | 49.1 ± 22.7 | 51.1 ± 20.3 | 48.7 ± 13.6 |
|  | ***CF/ECC*** | 50.6 ± 20.8 | 50.4 ± 18.3 | 55.2 ± 15.3 | 53.9 ± 18.6 | 53.3 ± 15.3 | 57.4 ± 17.3 |

BF-mid = 50% length of biceps femoris; BF-distal = 5 cm distal of BF-mid; ECC = eccentric training group; AF/ECC = acute fatigue + eccentric exercise; RF/ECC = residual fatigue +eccentric exercise.

**Table 4.** Representation of the changes in muscle stiffness for both measurement points.

|  |  | **Pre** | **Post** | **1h Post** | **24h Post** | **48h Post** | **72h Post** |
| --- | --- | --- | --- | --- | --- | --- | --- |
| **SWE BF-mid (kPa)** | ***ECC*** | 11.2 ± 2.5 | 13.4 ± 3.2 | 12.2 ± 2.1 | 12.9 ± 2.3 | 12.9 ± 2.9 | 12.9 ± 4.3 |
|  | ***AF/ECC*** | 9.5 ± 2.8 | 11.9 ± 4.0 | 11.7 ± 3.0 | 10.2 ± 3.0 | 10.8 ± 2.9 | 10.7 ± 3.0 |
|  | ***CF/ECC*** | 11.8 ± 2.1 | 12.4 ± 1.5 | 12.6 ± 2.9 | 11.3 ± 2.3 | 11.2 ± 2.8 | 10.3 ± 2.2 |
| **SWE BF-distal (kPa)** | ***ECC*** | 9.9 ± 2.7 | 11.7 ± 2.6 | 11.4 ± 3.3 | 10.6 ± 2.0 | 11.2 ± 2.4 | 10.1 ± 1.6 |
|  | ***AF/ECC*** | 9.5 ± 2.1 | 10.8 ± 4.7 | 11.6 ± 6.2 | 10.1 ± 2.3 | 11.3 ± 3.7 | 11.5 ± 3.4 |
|  | ***CF/ECC*** | 12.7 ± 3.0 | 13.6 ± 3.1 | 12.6 ± 2.4 | 13.1 ± 4.4 | 12.6 ± 4.0 | 12.5 ± 4.1 |

SWE = shear wave elastography; BF-mid = 50% length of biceps femoris; BF-distal = 5 cm distal of BF-mid; ECC = eccentric training group; AF/ECC = acute fatigue + eccentric exercise; RF/ECC = residual fatigue +eccentric exercise.
